# A non-viral DNA delivery system consisting of multifunctional chimeric peptide fused with zinc-finger protein

**DOI:** 10.1016/j.isci.2024.109464

**Published:** 2024-03-08

**Authors:** Siyuan Yu, Haifeng Pan, Han Yang, Haoyun Zhuang, Haihui Yang, Xuan Yu, Shiyin Zhang, Mujin Fang, Tingdong Li, Shengxiang Ge, Ningshao Xia

**Affiliations:** 1 State Key Laboratory of Vaccines for Infectious Diseases, Xiang An Biomedicine Laboratory, Department of Laboratory Medicine, School of Public Health, Xiamen University, Xiamen 361102, China; 2 National Institute of Diagnostics and Vaccine Development in Infectious Diseases, State Key Laboratory of Molecular Vaccinology and Molecular Diagnostics, Collaborative Innovation Center of Biologic Products, National Innovation Platform for Industry-Education Integration in Vaccine Research, NMPA Key Laboratory for Research and Evaluation of Infectious Disease Diagnostic Technology, the Research Unit of Frontier Technology of Structural Vaccinology of Chinese Academy of Medical Sciences, Xiamen University, Xiamen 361102, China

**Keywords:** Drug delivery system, Molecular biology

## Abstract

Non-viral gene delivery systems have received sustained attention as a promising alternative to viral vectors for disease treatment and prevention in recent years. Numerous methods have been developed to enhance gene uptake and delivery in the cytoplasm; however, due to technical difficulties and delivery efficiency, these systems still face challenges in a range of biological applications, especially *in vivo*. To alleviate this challenge, we devised a novel system for gene delivery based on a recombinant protein eTAT-ZF9-NLS, which consisted of a multifunctional chimeric peptide and a zinc-finger protein with sequence-specific DNA-binding activity. High transfection efficiency was observed in several mammalian cells after intracellular delivery of plasmid containing ZF9-binding sites mediated by eTAT-ZF9-NLS. Our new approach provides a novel transfection strategy and the transfection efficiency was confirmed both *in vitro* and *in vivo*, making it a preferential transfection reagent for possible gene therapy.

## Highlights

• eTAT-ZF9-NLS provides a novel transfection strategy based on a protein-DNA complex
• Plasmids can be efficiently transfected into several cell lines by eTAT-ZF9-NLS
• eTAT-ZF9-NLS shows good transfection performance under serum-rich conditions
• eTAT-ZF9-NLS has a potential to be designed as an *in vivo* DNA delivery agent

## Graphical abstract

**Figure:**
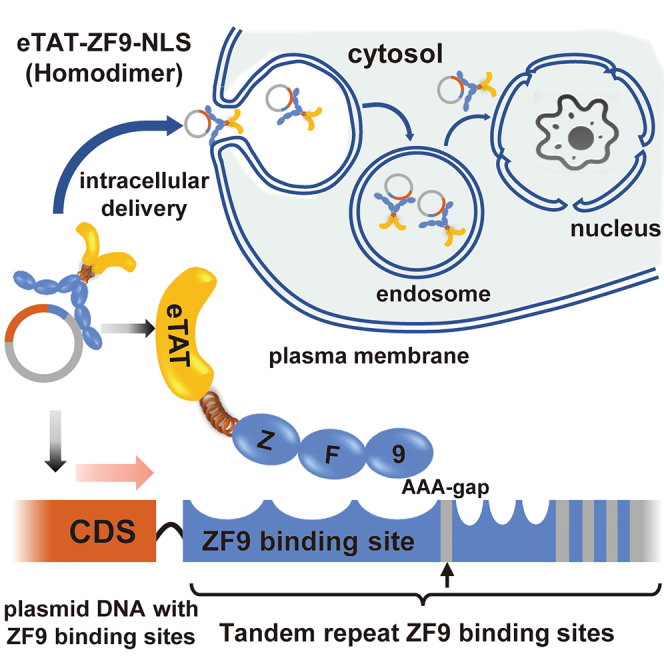

## Introduction

Transfection of DNA into mammalian cells is critical for many biological and medical studies.^1^ In recent years, numerous *in vivo* challenges that influence DNA transfection have been found gradually, such as nuclease degradation, non-specific binding with serum proteins, and genotoxicity.^2^^,^^3^^,^^4^ Virus vectors are most commonly used in gene therapy as a carrier due to their ability to carry genes efficiently and ensure long-term expression.^5^ However, the risk of provoking immune response^6^ and causing genome integration of viral DNA^7^ has restricted its practical application. Therefore, non-viral vectors have increasingly become a research hotspot in gene delivery, including cationic polymers and liposomes. The DNA molecules are entrapped within nanoparticles or lipid bilayers,^8^^,^^9^ which would lead to an aggregation state in the blood as a result of either colloidal instability or interaction with blood components, and thus affecting the expression of the delivered DNA.^10^ Overall, the gene delivery efficiency by existing non-viral strategies was significantly lower than those of viral vectors.^11^ Consequently, the *in vivo* transfection efficiency and safety of those carriers need to be further improved.

In recent years, a wide range of cell-penetrating peptides (CPPs), a series of short peptides capable of non-invasively mediating intracellular delivery of macromolecules with the advantages of low toxicity and no cell type restriction,^12^^,^^13^ has been used to overcome the bottlenecks of developing DNA-based therapeutics and toolkits.^14^^,^^15^ Examples of such peptides include the protein transduction domains (PTDs) of HIV-TAT,^16^^,^^17^ VP22,^18^ and polyarginine (R_n_),^19^^,^^20^ which have been shown to enhance DNA delivery *in vitro*, though CPPs alone does not enhance DNA uptake *in vivo*. Besides, it has been reported that DNA attached to proteins can avoid nuclease digestion, which has become another potential advantage of CPPs-based recombinant protein vectors in gene delivery. Although CPPs have many unique advantages, there are still several inherent flaws that affect their widespread use in biological research. Although many of these peptides can mediate efficient intracellular delivery of cargo, their endosomal escape and *in vivo* delivery efficiency are rather limited.^21^^,^^22^^,^^23^ It is essential to ensure efficient intracellular delivery, and the binding mode of CPPs to DNA is also a key factor affecting the successful expression of target genes. Currently, there are two primary approaches to delivering DNA cargo via CPPs: covalent binding and electrostatic interactions. However, the former presents a challenge due to the complex synthesis process required,^24^ while the latter may not be suitable for *in vivo* applications due to its limited stability and specificity. With the deepening of our knowledge of the issues, DNA-binding proteins are increasingly used as DNA delivery vehicles because of their ability to combine specific DNA sequences in some novel approaches.^25^^,^^26^^,^^27^ It was well known that Cys2His2 zinc-finger proteins (ZFs) could be used as artificial DNA-binding proteins which were found in a range of transcriptional regulatory proteins.^28^^,^^29^^,^^30^ Structural and functional characterization of the C2H2 ZF domains of ZFs makes it bind specifically to DNA sequences in three-base pairs.^31^

In previous work, based on TAT-PTD, we established a multifunctional chimeric peptide eTAT and demonstrated its enhanced delivery efficacy both *in vivo* and *in vitro.*^32^ With more effective endocytosis, higher serum tolerance, and the characteristics of promoting endosomal escape, it is speculated that the eTAT system has the potential for application in gene therapy. Based on the understanding of the shortcomings of the current gene delivery research progress in biological application, we designed a non-viral gene delivery strategy in which a recombinant protein was constructed by fusion of eTAT chimeric peptide with a series of ZF proteins to mediate the intracellular delivery of plasmids containing ZF-specific binding sites,^28^^,^^32^ and further investigated its capacity to mediate plasmid transfection both *in vitro* and *in vivo*.

## Results

### eTAT-ZF-NLS proteins enable transfection of their bound DNA

**Figure 1:**
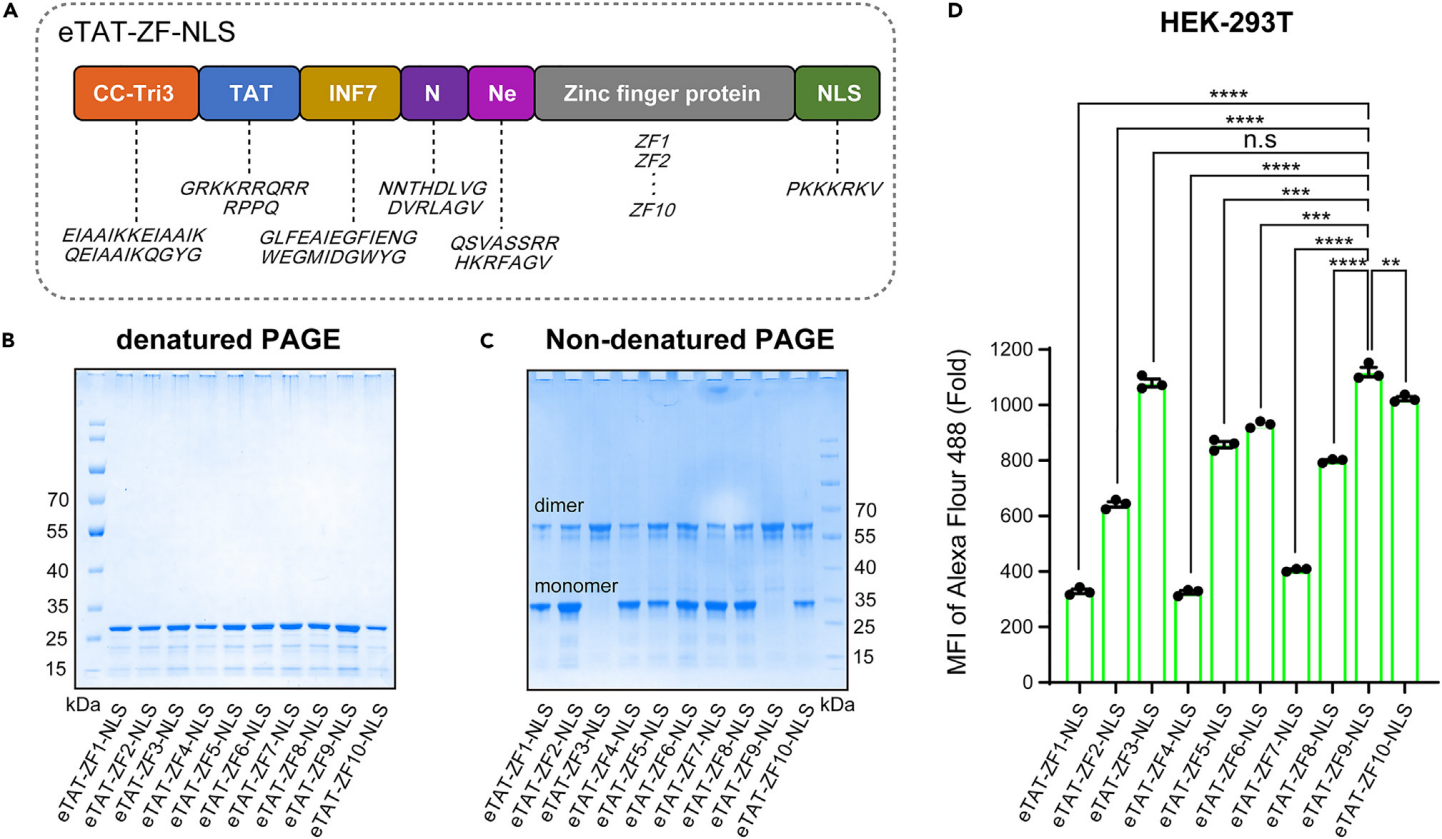
Series of eTAT-ZF-NLS protein for intracellular delivery (A) Schematic illustration of the series of eTAT-ZF-NLS recombinant protein, which is composed of ZF proteins derived from an OPEN ZF library (ZF1–ZF10) with an optimized eTAT chimeric peptide at the N terminus. (B) SDS-PAGE and (C) non-denaturing PAGE analysis of the purity and dimerization of eTAT-ZF-NLS proteins. (D) HEK293T cells were treated with eTAT-ZF-NLS series proteins at a final concentration of 2 μM for 24 h. After incubation and harvesting into single-cell suspensions, the cells were fixed with 4% paraformaldehyde and subsequently permeabilized with 0.3% Triton X-100. Anti-His tag antibody conjugated with Alexa 488 was used for analyzing the intracellular delivery efficiency of eTAT-ZF-NLS proteins by flow cytometry, different MFIs for the treated HEK293T cells show differences in ZF proteins on intracellular delivery. All the values are expressed as the means ± SEM, n = 3 for each group, and significance was determined by two-tailed Student’s t tests. ∗p < 0.05, ∗∗p < 0.01, ∗∗∗p < 0.001, ∗∗∗∗p < 0.0001, n.s: no significance.

The eTAT system is composed of the following four functional modules in the form of fusion expression, including TAT (T), a typical arginine-rich cell-penetrating peptide;^33^ INF7 (I), a pH-dependent membrane active peptide, which is mainly responsible for disrupting the endosomal membrane;^34^ two endosomal localized protease cleavage sites N (the cleavage site of cathepsin L) and Ne (the cleavage site of Furin),^35^ allowing cargos to be released from the endosomal membrane; and a leucine zipper (LZ),^36^ a kind of coiled coils, which helps increase the concentration of the local positive charge provided by TAT through self-dimerization, thereby improving the intracellular delivery efficiency of the system. To investigate whether eTAT combined with ZF protein can be used as DNA delivery vehicles, by referring to the ZF proteins library,^37^ tridactyl Cys2His2 ZFs specifically targeting a specific DNA sequence were employed. When the ZFs were fused to the original eTAT chimeric polypeptide, a serious chain break after purification was observed (Figure S1A). To solve this problem, we rearranged the positions of the elements in eTAT, and then replaced the LZ motif with another coiled-coil motif, CC-Tri3,^38^ to obtain an improved version of eTAT polypeptide suitable for delivering ZF series proteins. In addition, to make the protein-DNA complex enter the nucleus as much as possible after endosomal escape, we introduced a strong nuclear localization signal (NLS) SV40 NLS with the basic sequence of PKKKRKV, which was identified from the simian virus 40 (SV40) large-T antigen,^39^ and has been widely used to mediate protein entry into the nucleus; additional SV40 NLS fusion has been found to increase the intracellular activity of direct delivery of ZFN protein.^40^ The NLS was added at the C-terminal of the recombinant proteins (Figure 1A; Table S2). Thus, a series of improved eTAT-ZF-NLS recombinant proteins were prepared (Figure 1B). As analyzed by non-denaturing PAGE, all the eTAT-ZF-NLS proteins can form homodimers with different degrees (Figure 1C). Moreover, as analyzed by flow cytometry, the cellular uptake of TAT-ZF-NLS proteins in HEK293T cells is different, and the intracellular delivery efficiency was related to the degree of homodimerization, among which eTAT-ZF9-NLS exhibited the best effect (Figure 1D).

**Figure 2:**
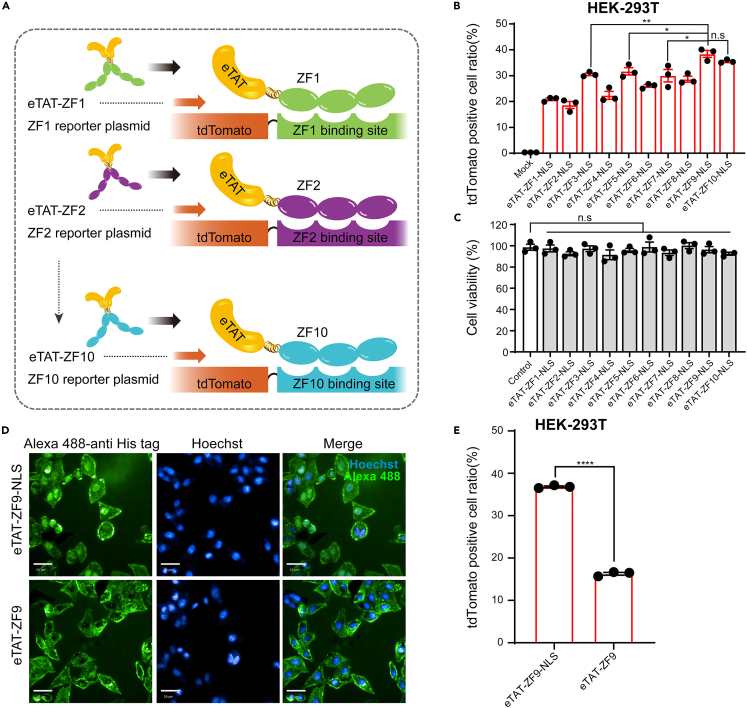
eTAT-ZF-NLS proteins bound to reporter plasmids used for *in vitro* transfection (A) Schematic of eTAT-ZF-NLS protein series and their corresponding fluorescent protein (tdTomato) reporter plasmids with ZF-binding sites to be bound. (B) eTAT-ZF-NLS protein series (2 μM) were mixed with reporter plasmids (10 μg) and delivered to HEK293T cells, after 48 h the tdTomato was expressed and the percentages of tdTomato-positive cells were plotted and analyzed using flow cytometry. (C) HEK293T cells were treated with eTAT-ZF-NLS proteins under the same conditions as with the previous experiments, then a CCK-8 assay was performed. The column represented the percentages of cell viability. (D) Representative fluorescence microscopic images (magnification, ×200) acquired with the Phenix Opera system showed the distribution of intracellular green fluorescence of eTAT-ZF-NLS and eTAT-ZF-treated HeLa cells stained with Alexa Flour 488 labeled anti-His tag antibody. Nuclear material (Hoechst 33342, blue), eTAT-ZF9-NLS (Alexa Flour 488, green). All scale bars denote 50 μm. (E) Flow cytometry analysis measured the percentages of tdTomato-expressing HEK293 cells after 48 h post-transfection with eTAT-ZF9-NLS or eTAT-ZF9 (no NLS)-bounded reporter plasmid, respectively. For B, C, and E, all values are expressed as the means ± SEM (n = 3), and significance was determined by two-tailed Student’s t tests. ∗p < 0.05, ∗∗p < 0.01, ∗∗∗∗p < 0.0001, n.s: no significance.

After verifying that the eTAT-ZF-NLS recombinant proteins can penetrate into HEK293T cells, a series of red fluorescent protein, tdTomato-expression reporter plasmids containing variants of 9-bp ZF proteins binding site (2gap) (Table S1) was constructed for evaluating DNA transfection efficiency by binding eTAT-ZF-NLS proteins, respectively (Figure 2A). The plasmid was selected as the DNA vector because it was shown that the transfection efficiency was higher than the linear DNA obtained by PCR amplification.^41^ For this experiment, the reporter plasmid was transfected into HEK293T cells with different eTAT-ZF-NLS proteins. 48 h post-transfection, cells were harvested and intracellular tdTomato signals were analyzed by flow cytometry. The result showed that eTAT-ZF9-NLS exhibited the highest transfection efficiency (38%), followed by eTAT-ZF10-NLS, which were significantly higher than eTAT-ZF3-NLS, eTAT-ZF5-NLS, or eTAT-ZF7-NLS (Figure 2B). A good cytocompatibility is the basic requirement for the development of a novel transfection method, so we further measured the cell viabilities after eTAT-ZF-NLS treatment by using Cell Counting Kit-8 (CCK-8) assay. As a control, cells were treated with an equal volume of protein storage buffer. It was apparent that after 48 h, eTAT-ZF-NLS-mediated DNA transfection did not cause significant cytotoxicity as compared to the control group, respectively (Figure 2C). In addition, to investigate the contribution of NLS in this system, eTAT-ZF9 (no NLS) recombinant protein was prepared (Figure S1B) and compared with the efficiency of eTAT-ZF9-NLS to deliver the reporter plasmid. Anti-His tag antibody coupled to Alexa 488 was used and obvious nuclear localization was observed after intracellular delivery of eTAT-ZF9-NLS in HeLa cells, but not in eTAT-ZF9 (no NLS) group (Figure 2D), Flow cytometry results showed that after removal of NLS, the percentage of tdTomato-positive cells sharply decreased to 16% under the same transfection conditions in HEK293T cells (Figure 2E). These results suggested that eTAT-ZF9-NLS-mediated plasmid delivery can actively transport into the nucleus after entering the cell, which may make this delivery system an advantage over conventional transfection reagents in DNA transfection.

### Numbers of binding sites affect eTAT-ZF9-NLS-mediated transfection efficiency

**Figure 3:**
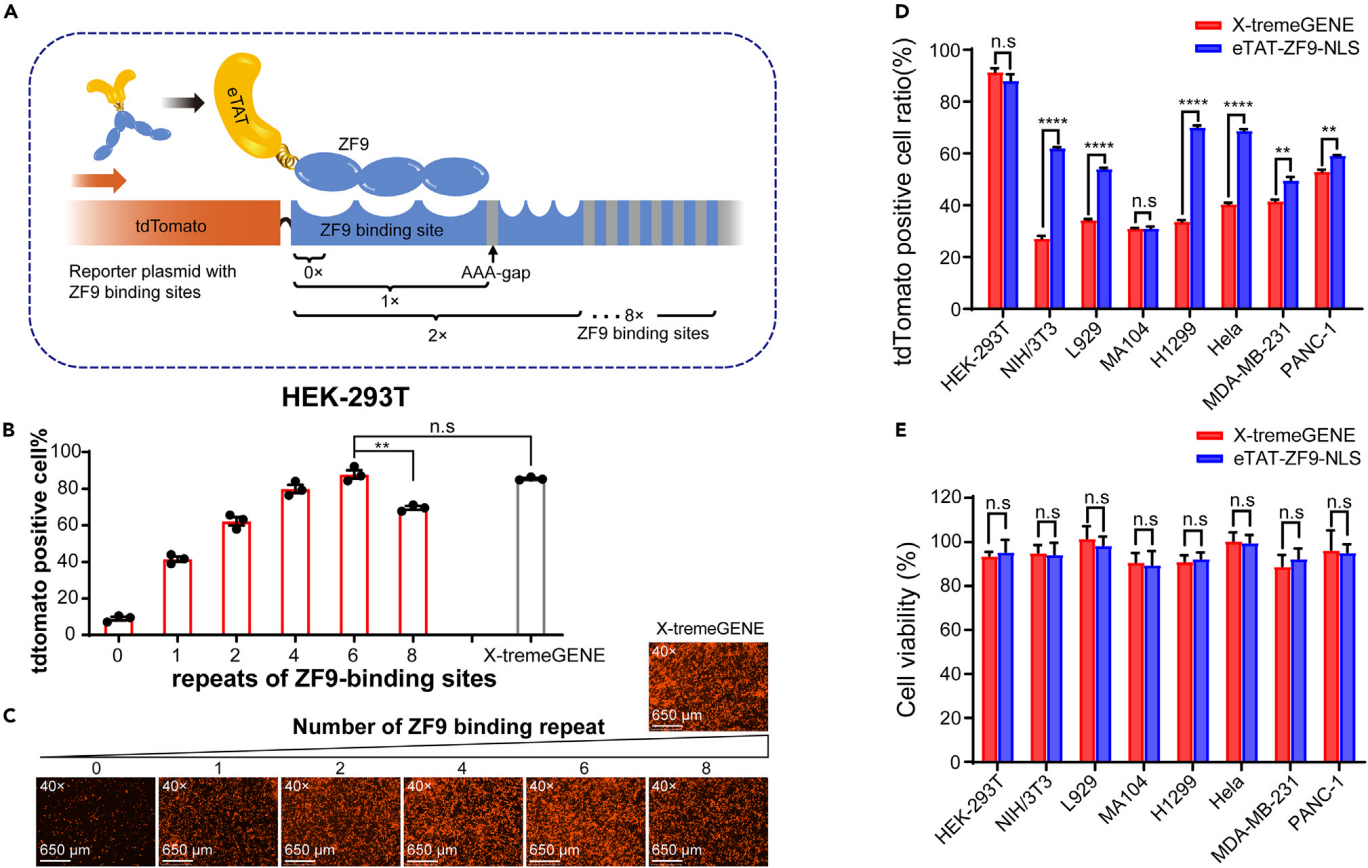
Effect of binding site numbers within the reporter plasmid on eTAT-ZF9-NLS-mediated transfection eTAT-ZF9-NLS (2 μM) mixed with fluorescent protein (tdTomato) reporter plasmids containing 0×, 1×, 2×, 4×, 6×, or 8× tandem repeats of ZF9-binding sites (10 μg), respectively, and transfected into HEK293T cells, the schematic as described in (A). After 48 h of transfection, (B) the percentages of tdTomato expression cells were analyzed by flow cytometry, (C) and the intracellular expression of tdTomato was observed by fluorescence microscopy. (D) Different cell lines were transfected with tdTomato reporter plasmids using eTAT-ZF9-NLS and X-tremeGENE methods, respectively. The percentages of tdTomato-positive of the selected cell lines were assessed 48 h after transfection by flow cytometry. (E) Different cell lines were transfected with reporter plasmids using eTAT-ZF9-NLS and X-tremeGENE for 48 h, then the culture mediums were collected and the cell viabilities were assessed by performing CCK-8 assay. The percentages of cell viability were shown in the column. For B, D, and E, values are expressed as the means ± SEM (n = 3), Significance determined by Student’s *t* test, ∗∗p < 0.01, ∗∗∗∗p < 0.0001, n.s: no significance, and the commercial transfection reagent X-tremeGENE was used as a control in B, C, D and E.

Since the eTAT-ZF9-NLS displayed considerable plasmid DNA transfection efficiency, we chose it for further characterization. It is speculated that the copies of the ZF9-binding site might be related to the ZF9-binding affinity to the reporter plasmid, thus further affecting the eTAT-ZF9-ZLS-mediated DNA transfection efficiency. To test this hypothesis, tdTomato reporter plasmids containing 0×, 1×, 2×, 4×, 6×, or 8× tandem repeats of the ZF9-binding site were constructed and used for intracellular delivery of HEK293T cells mediated by eTAT-ZF9-NLS (Figure 3A). Then the ratio of tdTomato-positive cells was analyzed by flow cytometry. The results showed that when the copies of the tandem repeat of the ZF9-binding site increased from 0 to 6, the transfection efficiency increased significantly from 9% to 88%. However, when the tandem repeats increased to 8×, the transfection efficiency decreased (Figure 3B). The fluorescence microscopy imaging showed the same results (Figure 3C). Agarose gel electrophoresis analysis also showed that the increase in the number of ZF9-binding sites provided a more sufficient binding of pDNA to protein (Figure S3). In addition, the transfection efficiency of eTAT-ZF9-NLS under optimal conditions was comparable to that of the commercial DNA transfection reagent X-tremeGENE when transfection was conducted in serum-free medium. Based on the obtained experimental results, we finally determined 6 copies of the binding site to establish the transfection system.

To determine whether the transfection strategy has universal applicability, transfection experiments were performed on different cell lines including tumor cells and normal cells. 48 h post-transfection, the tdTomato-positive cell ratios were analyzed by flow cytometry. The results of the bar graph showed that eTAT-ZF9-NLS showed significantly higher transfection efficiency than the control transfection reagent in both tumor cells and normal cells (Figure 3D). Next, from the CCK-8 assay, it can be seen that under the same experimental condition, the viabilities of the aforementioned cells were not significantly affected by eTAT-ZF9-NLS-mediated transfection (Figure 3E). We also measured the IC50 values of eTAT-ZF9-NLS in several representative mammalian cells to further confirm its cytotoxicity; the results showed all the detected cells exhibited relatively low cytotoxicity (Figure S5) with IC50 > 40 μM, which were more than 20-fold higher than the concentration used for actual transfection of the plasmids (2 μM).

### eTAT-ZF9-NLS-mediated plasmid transfection demonstrates serum tolerance

**Figure 4:**
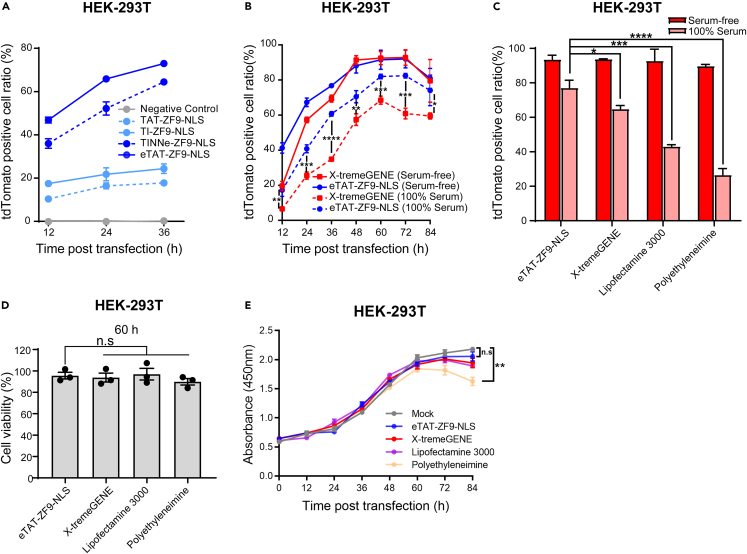
eTAT-ZF9-NLS has advantages *in vitro* plasmid transfection, especially under serum conditions (A) The expression of tdTomato in the HEK293T cells transfected with different ZF9-NLS-related recombinant proteins as indicated. HEK293T cells were treated with a mixture of recombinant ZF9-related proteins (2 μM) and the tdTomato expression plasmid (10 μg) for 3 h and then washed. Intracellular tdTomato signals were analyzed by flow cytometry every 12 h. The differences between the eTAT-ZF9-NLS group and the TINNe-ZF9-NLS group are indicated. (B) The expression of tdTomato in the HEK293T cells transfected with eTAT-ZF9-NLS and X-tremeGENE. The cells were transfected in the absence of FBS or in the presence of 100% FBS, and the intracellular tdTomato signals were analyzed by flow cytometry every 12 h. The differences between the eTAT-ZF9-NLS group and the X-tremeGENE group under the same transfection conditions are indicated. (C) The HEK293T cells were transfected with tdTomato overexpression plasmid in serum-free or serum conditions using various transfection reagents (eTAT-ZF9-NLS, X-tremeGENE, Lipofectamine 3000, and polyethyleneimine). The cells were harvested 60 h post-transfection and the ratio of tdTomato-positive cells under each transfection condition was analyzed by flow cytometry. (D) A CCK-8 assay was used to detect the cytotoxicities of cells transfected by the above four methods at 60 h in serum-free conditions, respectively. The results were shown in column. (E) The cell growth viabilities were presented as a cell proliferation curve. Significance for B, C, D, and E determined by Student’s *t* test, ∗p < 0.05, ∗∗p < 0.01, ∗∗∗p < 0.001, ∗∗∗∗p < 0.0001, n.s: no significance.

**Figure 5:**
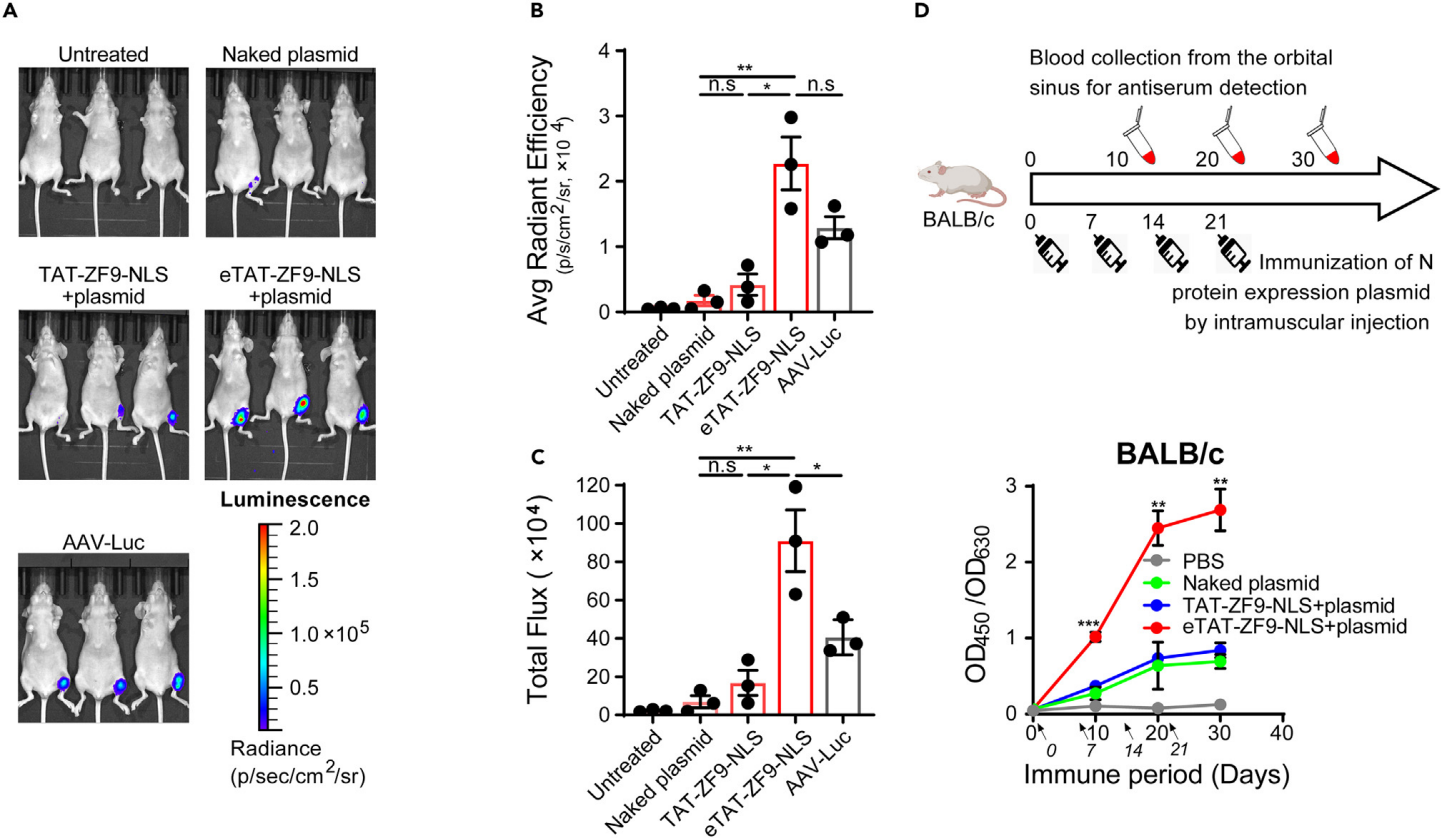
eTAT-ZF9-NLS-mediated efficient transfection *in vivo* The luciferase reporter plasmid (20 μg) was delivered to nude BALB/c nude mice by intramuscular injection of the hind leg mixed with TAT-ZF9-NLS (5 nmol) or mixed with eTAT-ZF9-NLS (5 nmol), respectively, the same amount of naked plasmid injection group was used as a negative control. For the transfection standard approach, mice were injected with 10 μL of luciferase-expressing AAV vector with a particle titer of 1×10^12^/mL. 48 h after injection, D-luciferin (150 mg/kg) was injected intraperitoneally. (A) The expression of luciferase was monitored by the live-animal imaging system, and the region of interest (ROI) of the hind leg was measured and analyzed by (B) average radiant efficiency and (C) total flux (n = 3). (D) BALB/c nude mice were immunized by intramuscular injection of 20 μg SARS-CoV-2 nucleocapsid protein expression plasmid via different delivery methods once every week, for a total of four times (n = 3). The antibody responses against SARS-CoV-2 nucleocapsid protein were detected by indirect binding ELISA using the sera collected from the orbital vein at 10, 20, or 30 days after the first immunization. The data in B, C, and D are expressed as the means ± SEM, significances were determined by Student’s *t* test, and p value are shown, ∗p < 0.05, ∗∗p < 0.01, ∗∗∗p < 0.001, n.s: no significant difference.

Before evaluating the serum tolerance of eTAT-ZF9-NLS, we confirmed the contribution of INF7, NNe, and LZ in the eTAT system to mediated target DNA delivery. The recombinant proteins TAT-ZF9-NLS (T-ZF9-NLS), TAT-INF7-ZF9-NLS (TI-ZF9-NLS), and TAT-INF7-NNe-ZF9-NLS (TINNe-ZF9-ZLS) (Table S3) were expressed and purified from *E. coli* (Figure S1C). Then tdTomato reporter plasmid containing 6 copies of the ZF9-binding site was transfected with different recombinant ZF9 proteins, respectively. Flow cytometry results showed that eTAT-ZF9-NLS exhibited the highest transfection efficiency at each transfection time point, followed by TINNe-ZF9-NLS, both of which were significantly higher than TI-ZF9-NLS or T-ZF9-NLS (Figure 4A). Then, we explored the efficiency of delivery *in vivo* conditions, which was simulated by FBS. The eTAT-ZF9-NLS and tdTomato reporter plasmids were mixed and transfected into HEK293T cells cultured in 100% FBS and serum-free medium, respectively. The cells were harvested at different time points post-transfection and the ratio of tdTomato-positive cells was analyzed by flow cytometry. Under serum-free conditions, the ratio of tdTomato-positive cells in the eTAT-ZF9-NLS group remained higher than that in the X-tremeGENE group through 12–36 h post-transfection, suggesting that eTAT-ZF9-NLS could mediate plasmid DNA entry and expression more rapidly (Figure 4B). From 48 h post-transfection until the end of the time course, the efficiency remained similar in the two experimental groups. Nevertheless, under the condition of 100% FBS, the transfection efficiency of the eTAT-ZF9-NLS group maintained a relatively high level. Although X-tremeGENE was reported to have high transfection efficiency in the serum-containing medium,^42^ its transfection efficiency in the presence of 100% serum was significantly lower than that of eTAT-ZF9-NLS (Figure 4B). In the comparison of various transfection reagents (X-tremeGENE, Lipofectamine 3000, and polyethyleneimine), the transfection efficiency of eTAT-ZF9-NLS was comparable or higher than other transfection reagents under serum-free conditions and also shows the best performance under serum conditions (Figure 4C). Cell viabilities under four transfection reagents were also determined by CCK-8 assay; the result showed that no additional negative effect was caused on cell viability after 60 h transfection mediated by eTAT-ZF9-NLS, compared to other widely used commercial transfection reagents (Figure 4D). To explore the reason for the decrease in the ratio of positive cells after 60 h transfection (Figure 4B), we further extended the time point of CCK-8 detection to 84 h. According to the cell growth curve, it was observed that a significant decrease in cell viability occurred at the later stage post-transfection, which was severe in the polyethyleneimine group, while slightly in the eTAT-ZF9-NLS group (Figure 5E).

### eTAT-ZF9-NLS-mediated efficient plasmid delivery and expression *in vivo*

After confirming that eTAT-ZF9-NLS-mediated DNA delivery exhibits excellent serum resistance, we explored the feasibility of the system for *in vivo* DNA delivery. The firefly luciferase reporter system is widely used in *in vivo* bioluminescence imaging due to its high sensitivity and stability. Based on this system, a luciferase expression plasmid containing 6 copies of the ZF9-binding site was constructed and mixed with different ZF9 recombinant proteins; after that, the mixtures were administered into BALB/c intramuscularly into the left hind leg and the same amount of naked plasmid used as the negative control. Because the adeno-associated virus (AAV) vector is widely used for stable gene transfer *in vivo* and provides efficient systemic gene delivery directly to skeletal muscle,^43^^,^^44^ we used an AAV-expressing luciferase reporter (AAV-Luc) as a standard control. When measured 48 h post-injection, the administration of eTAT-ZF9-NLS/plasmid resulted in the strongest luciferase activity in the left hind leg of mice observed by the live-animal imaging system (Figure 5A). In the TAT-ZF9-NLS/plasmid group with the same dose of eTAT, only two of the three mice were observed with obvious luciferase signals but were significantly weaker than the eTAT group. While almost no activity was observed in the mice injected with the naked plasmid or in the untreated group. Then, regions of interest were delineated manually on the left hind leg of mice, and their total fluorescence intensity was readout. The results showed the fluorescence intensity of eTAT group was approximately 5-fold higher than that obtained from TAT group and more than 2-fold higher than the AAV-Luc group (Figures 5B and 5C).

To investigate whether eTAT-ZF9-NLS-mediated DNA delivery *in vivo* has the potential of becoming a promising efficient strategy for DNA immunization and antigen screening methods, the ZF9 recombinant proteins mixed with the severe acute respiratory syndrome coronavirus 2 nucleocapsid protein-encoding plasmid were used to induce an immunization in mice by intramuscular injection. The antiserum was 1,000-fold diluted and detected by indirect ELISA after three-dose immunization. The results showed that eTAT-ZF-NLS-mediated DNA intramuscular immunization induced a significantly stronger immune response than other treatment groups, while immunization with plasmid mixed with TAT-ZF9-NLS did not induce specific immune responses (Figure 5D), which was consistent with the previous results.

**Figure 6:**
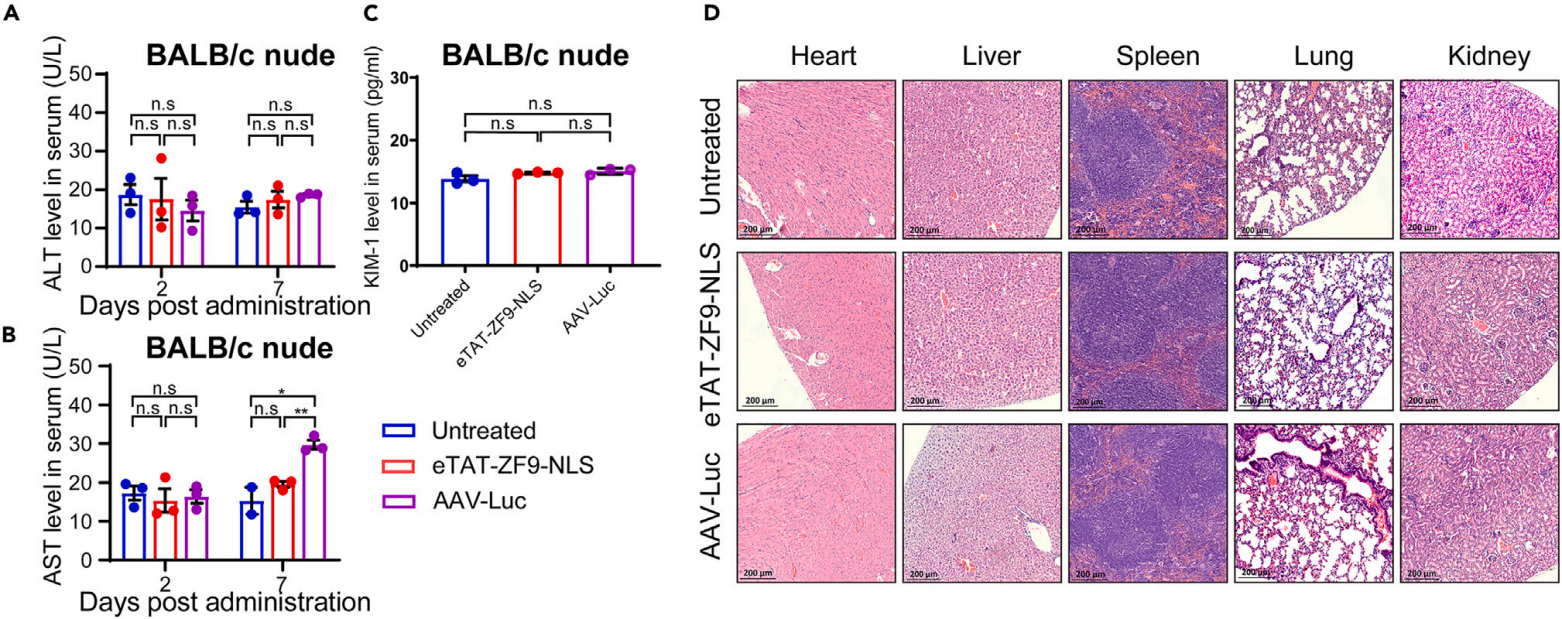
The biosafety evaluation of eTAT-ZF9-NLS *in vivo* Serum liver damage marker (A) aminotransferase (ALT) and (B) aminotransferase (AST) levels, and a renal injury marker (C) kidney injury molecule-1 (KIM-1) (C) levels were detected after eTAT-ZF9-NLS administration. Sera were collected by retro-orbital bleeding from each mouse. (D) H&E staining for heart, liver, spleen, lung, and kidney in each group (magnification, 200×). The data in A, B, and C are expressed as the means ± SEM, significances were determined by Student’s *t* test, and p value are shown, ∗p < 0.05, n.s: no significant difference.

Low *in vivo* toxicity is also one of the key factors determining whether a DNA delivery system has the potential for future clinical application. Therefore, some toxicity evaluations were carried out. We confirmed the biosafety of eTAT-ZF9-NLS treatment through comprehensive experiments and used AAV as a control. Serum aminotransferase (ALT) and aminotransferase (AST) were selected as parameters for the evaluation of hepatotoxicity, and kidney injury molecule-1 (KIM-1) was used for the evaluation of renal toxicity. We measured serum ALT and AST levels after 2 and 7 days of administration and serum KIM-1 levels after 7 days. Compared with the untreated groups, there was no significant increase in the serum levels of ALT (Figure 6A), AST (Figure 6B), and KIM-1 (Figure 6C) observed in eTAT-ZF9-NLS-mediated transfected mice at 2 and 7 days post-administration. However, the serum AST value of the AAV-Luc group after 7 days of administration showed a slight significant increase, but it was still within the normal range (<40 U/L) (Figure 6B). Moreover, H&E staining of major organs (including heart, liver, spleen, lung, and kidney) also showed no obvious tissue damage after 7 days of eTAT-ZF9-NLS administration (Figure 6D). The aforementioned results indicated that eTAT-ZF9-NLS does not exhibit detectable systemic toxicity within the dose range used in this study; thus, it may be compatible with *in vivo* applications.

## Discussion

In this study, we successfully achieved efficient DNA delivery by using the eTAT system involving a DNA-binding domain, ZF9, which showed high efficiency in a variety of cell lines and high tolerance to serum conditions. Also, it was found that eTAT-ZF9-NLS obtained a better efficiency than AAV *in vivo* transfection in a short period of time, which verified the feasibility of the eTAT system for nucleic acid delivery by fusion expression of nucleic acid-binding proteins. It is foreseeable that eTAT manifests the potential to transfect nucleic acids, such as plasmids or mRNAs, or to transfect a complex of functional proteins and nucleic acids, such as the CRISPR-Cas9 sgRNA complex,^45^^,^^46^ *in vitro* and *in vivo*.

pDNA transfection abilities of eTAT system were strongly dependent on the delivery efficiency of DNA-binding proteins. Among the eTAT-ZF-NLS series proteins, ZF3, ZF9, and ZF10 had significantly higher intracellular levels at the same delivery concentration (Figure 1D). Correspondingly, the transfection efficiency of the reporter gene of pDNA to be delivered by the aforementioned three proteins was also significantly higher than that of other proteins in the series (Figure 2B). This phenomenon seems to be related to the degree of dimerization of these recombinant proteins. We observed in non-denaturing electrophoresis analysis that ZF3 and ZF9 exist entirely in the form of dimers, but ZF10, which also mediates high transfection efficiency, still exists in the form of partial monomers (Figure 1C). Although obtained results fully indicated that the ZF-binding site was necessary for the donor DNA, the position of the binding site in the donor DNA, the form of DNA (linear, circular, or fragmented), remained to be explored, and the limitations of eTAT-ZF-NLS on the sort of plasmid, the degree of the double helix structure, and the molecular weight of the carried DNA still needed to be further studied. Moreover, the intracellular location of plasmid DNA, such as nuclear localization, is also an important factor in determining the transfection efficiency,^47^ which was also confirmed in this study (Figure 2E). Although recent works have pointed out that the tandem repeat of NLS can improve the efficiency of gene editing,^48^ it is unclear whether it also has a significant effect on gene transfection; future research should explore this issue. Above all, to gain further performance of the intracellular expression abilities of CPP-mediated DNA transfection, in addition to improving the intracellular internalization efficiency of CPPs, the binding affinity of the delivery system to DNA and the ability to enter the nucleus can be used as the focus of optimization.

Efficient DNA transfection, especially *in vivo* transfection, is critical for biological research.^49^ Unfortunately, although great progress has been made in the research of DNA delivery technology, the current *in vivo* DNA delivery efficiency is still poor.^50^ Even though CPPs have been shown to have the advantage of selectively crossing the blood-brain barrier,^51^ CPP-based delivery is also challenged by serum tolerance, a phenomenon in which serum concentration sharply decreases successful delivery,^52^^,^^53^ which severely limits its application in situations in which serum is unavoidable, particularly *in vivo*. In the previous study, the transduction activity in serum condition of several recombinant proteins was significantly improved by the formation of homodimer induced by the hydrophobic interaction controlled by a coiled coil.^32^ However, it is worth noting that the improvement degrees in the delivery ability of various cargo proteins were also different when mediated by the eTAT system. In this study, after identifying ZF9 as the DNA-binding protein for our eTAT-based DNA delivery strategy, we also considered whether the delivery via eTAT-ZF9-NLS could overcome the serum tolerance. The results showed that even in 100% serum conditions, the positive cell ratio of the target gene was still approximately 80% compared to no serum conditions (Figures 4B and 4C). Moreover, the serum tolerance of eTAT-ZF9-NLS was significantly higher than that of the commonly used transfection reagent X-TremeGENE (Figure 4C). These results suggested the feasibility of *in vivo* delivery via eTAT-ZF9-NLS, which has been demonstrated in mice (Figure 5).

Transfection efficiency is an important issue in the application of gene therapy, and it is also the main limiting factor affecting the clinical transformation of existing DNA delivery systems.^54^ Several evidences have shown that the transfection efficiency of genes for different types of somatic cells is significantly different,^55^^,^^56^ especially the expression level of exogenous genes in terminally differentiated cells is relatively low.^57^ However, the advantage of eTAT-ZF9-NLS in the transfection efficiency of different cells under serum conditions and *in vivo* transfection of muscle cells seems to indicate the difference in performance between eTAT-ZF9-NLS and other traditional transfection methods. Future efforts on this point may be helpful to the understanding of requirement of *in vivo* transfection. Another advantage of this system is that the short retention time of the target protein may indicate its advantage of low genotoxicity and fewer genetic risks^58^ (Figure S4). Hence, it can be regarded as an attractive DNA-mediated administration method and has a promising clinical application prospect, which makes the system a promising therapeutic tool for rare diseases caused by defects in a single gene or multiple genetic mutations such as acute tyrosinemia and myasthenia gravis. Not only that but delivering the correct gene sequence to intervene in the disease can also avoid the inefficient and off-target problems caused by gene-editing technology. In addition, the system also shows the potential as a DNA vaccine. Although mRNA vaccines have shown strong immune responses in clinical trials, however, DNA vaccines also have many unique advantages such as easy production, more stable product than mRNA vaccines, easier storage,^59^ and induction of a large number of T cell responses.^60^ In this study, we demonstrated a highly immune effect was induced by eTAT-ZF9-NLS-mediated gene immunization in mice (Figure 5D), which makes the eTAT-ZF9-NLS system a potential non-viral DNA delivery strategy of vaccine candidates.

In summary, the main innovations and achievements of this study are as follows. First, the DNA-binding protein fused with eTAT described here presents a novel concept for the design of a CPP-based DNA transduction agent. Second, eTAT-ZF9-NLS has been demonstrated to have an efficient ability to mediate DNA delivery into somatic cells *in vivo* with the potential to be designed as a non-viral *in vivo* DNA delivery system. Future research should be undertaken to explore the actual effect of the strategy in the prevention and treatment of infectious and genetic diseases.

### Limitations of the study

There are still several limitations that should be considered. The factors affecting transfection efficiency other than the penetration of CPPs and the binding force of ZF to DNA, such as the molecular weight of pDNA and the intracellular stability of protein-DNA complexes, remain to be explored. Not only that, the eTAT fusion proteins were expressed by a prokaryotic expression system; there may exist some difference in global conformations compared to the native proteins from eukaryotic sources; even some hydrophobic DNA-binding proteins are difficult to express and purify. The problems limit the choice of better alternatives to ZF9 protein. Besides, targeting modules or protective elements should be added to the system for targeted delivery *in vivo*, and further investigations should be carried out to express the target gene in various tissues and organs to further expand the application potential for gene therapy and other *in vivo* transfection capabilities.

## STAR★Methods

### Key resources table

**Table:** 

REAGENT or RESOURCE	SOURCE	IDENTIFIER
**Antibodies**		

Alexa Fluor(R) 488 anti-His tag	Abcam	Cat#362607; RRID: AB_2734397

**Bacterial and virus strains**		

AAV-Luc	GenScript Biotech Corporation	N/A

**Chemicals, peptides, and recombinant proteins**		

XtremeGENE	Roche	Cat#06366236001
Lipofectamine 3000	Invitrigen	Cat#L3000015
polyethyleneimine	YEASEN	Cat#40816ES03
eTAT-ZF1-NLS protein	This paper	N/A
eTAT-ZF2-NLS protein	This paper	N/A
eTAT-ZF3-NLS protein	This paper	N/A
eTAT-ZF4-NLS protein	This paper	N/A
eTAT-ZF5-NLS protein	This paper	N/A
eTAT-ZF6-NLS protein	This paper	N/A
eTAT-ZF7-NLS protein	This paper	N/A
eTAT-ZF8-NLS protein	This paper	N/A
eTAT-ZF9-NLS protein	This paper	N/A
eTAT-ZF10-NLS protein	This paper	N/A
T-ZF9-NLS protein	This paper	N/A
TI-ZF9-MLS protein	This paper	N/A
TINNe-ZF9-NLS protein	This paper	N/A
eTAT-ZF9 protein	This paper	N/A
Hoechst 33342	Thermo Fisher	Cat#H3570
Luciferin	Promega	Cat#P1042

**Critical commercial assays**		

Gibson assembly master mix	NEB	Cat#E2611L
ProteinIso Ni-NTA Resin	TransGen Biotech	Cat#DP101-02
Cell Counting Kit-8	MCE	Cat#HY-K0301
ALT assay kit	Jiancheng	Cat#C009-2-1
AST assay kit	Jiancheng	Cat#C010-2-1
Mouse KIM-1 ELISA kit	Beyotime	Cat#PK682

**Experimental models: Cell lines**		

HEK-293T	Han’s lab	Cat#CRL-3216; RRID: CVCL_0063
MA104	ATCC	Cat#CRL2378.1; RRID: CVCL_3845
L929	ATCC	Cat#CCL-1; RRID: CVCL_IW05
NIH/3T3	ATCC	Cat#CRL-1658; RRID: CVCL_0594
NCI-H1299	ATCC	Cat#CRL-5803; RRID: CVCL_0060
Hela	ATCC	Cat#CRM-CCL-2; RRID: CVCL_0030
MDA-MB-231	ATCC	Cat#CRM-HTB-26; RRID: CVCL_0062
PANC-1	ATCC	Cat#CRL-1469; RRID: CVCL_0480

**Experimental models: Organisms/strains**		

BALB/c nude mice	SLAC Laboratory Animal Co., Ltd	N/A
BALB/c mice	SLAC Laboratory Animal Co., Ltd	N/A

**Recombinant DNA**		

pLVX-TetOne-Puro-Luc	Clontech	Cat#631849
pET-eTAT-ZF1∼10-NLS	This paper	N/A
pET-T-ZF9-NLS	This paper	N/A
pET-TI-ZF9-NLS	This paper	N/A
pET-TINNe-ZF9-NLS	This paper	N/A
pET-eTAT-ZF9	This paper	N/A
pTT5-ZF1∼10-binding-tdTomato	This paper	N/A
pTT5-ZF9-binding-Luciferase	This paper	N/A
SARS-CoV-2 nucleocapsid protein-encoding plasmid	SinoBiological	Cat#40588-V08B

**Software and algorithms**		

FlowJo (version 10)	FlowJo	https://www.flowjo.com/
Living Image software	PerkinElmer	https://www.perkinelmer.com/
ZEN 3.4 (blue edition)	Zeiss	https://www.zeiss.com/
GraphPad Prism 7	GraphPad	https://www.graphpad.com/

### Resource availability

#### Lead contact

Further information and requests regarding resources, data, and reagents described in this manuscript should be directed to the lead contact, Dr. Shengxiang Ge (sxge@xmu.edu.cn).

#### Materials availability

All unique/stable reagents generated in this study are available from the lead contact with a completed Materials Transfer Agreement.

#### Data and code availability

• All data reported in this paper will be shared by the lead contact upon reasonable request.
• The original code is not reported in this paper.
• Any additional information required to reanalyze the data reported in this paper is available from the lead contact upon request.

### Experimental model and study participant details

#### Cell lines

HEK-293T human embryonic kidney cell line (ATCC, CRL-3216) was generously provided by Professor Jiahuai Han in Xiamen University, MA-104 embryonic rhesus monkey kidney cell line (CRL2378.1), L-929 mouse fibroblast (CCL-1), NIH/3T3 mouse fibroblast (CRL-1658), NCI-H1299 human non-small cell lung carcinoma cell (CRL-5803), Hela human cervix carcinoma cell (CRM-CCL-2), MDA-MB-231 human adenocarcinoma cell (CRM-HTB-26), and PANC-1 human epithelioid carcinoma cell (CRL-1469) were purchased from ATCC (ATCC, Virginia, USA), all of which were cultured in high-glucose DMEM medium supplemented with 10% FBS and 1% penicillin/streptomycin antibiotics (Thermo Fisher) at 37°C in a humidified incubator with 5% CO_2_.

#### Animals

BALB/c and BALB/c nude mice were supplied from the Shanghai SLAC Laboratory Animal Co., Ltd. The animals were housed in individual ventilated cages (IVCs) and kept under standard conditions with unlimited access to food and water on a 12/12 h light/dark cycle (lights on from 07:00 to 19:00 h), at a temperature of 22∼25°C and a relative humidity of 45∼55%.

#### Ethics statement

All animal experiments were carried out according to the guidelines of laboratory animals in China with the regulations of the Animal Welfare and Ethics Committee at Xiamen University (XMULAC20220276). Animal suffering was minimized or prevented at all times to improve their welfare.

### Method details

#### Expression and purification of the recombinant proteins

eTAT-ZF1∼ZF10-NLS and other CPP-ZF9-NLS prokaryotic expression plasmids derived from the pET-21b (+) vector were transformed into Escherichia coli BL21 (DE3) cells (New England Biolabs), and protein expression was induced with 1 mM isopropyl-β-D-thiogalactopyranoside (IPTG) administered at 25°C for 8 h. The cells were harvested and resuspended in lysis buffer containing 30 mM Tris-HCl buffer (pH 6.8) and 1% Triton X-100. After cell lysis by ultrasonication and high-speed centrifugation at 25,000×g for 20 min, the supernatant was filtered through a 0.45 μm microporous membrane. Subsequently, the His-tag-containing proteins were purified via Ni-NTA affinity chromatography. The imidazole was removed via dialysis in storage buffer containing 30 mM Tris-HCl buffer (pH 6.8) and 5% Glycerol. At last, the concentration of purified protein was measured using a Bradford assay, and aliquots were stored at –80°C until use.

#### Cell viability assays

Cell viability was tested by Cell Counting Kit-8 (CCK-8, MCE, New Jersey, USA) following the manufacturer’s protocols. Cells were seeded (1×10^4^ cells/well) in 96-well plates and treated with corresponding transfection reagents at different concentrations for the desired transfection time, respectively. The previous medium was then replaced with 90 μL of fresh free medium and 10 μL of CCK-8 solution and then incubated for another 30 minutes. Finally, the absorbance values (OD) at 450 nm were obtained using a Multiskan Go microplate spectrophotometer (Thermo Fisher). Cell viability was calculated using the following formula: Cell viability (%) = [(absorbance of test well - absorbance of blank well) / (absorbance of control well − absorbance of blank well)] × 100. The value of half-maximal inhibitory concentration (IC50) was determined from the inhibition concentration dependence evaluation according to a dose vs. response curve. Cell inhibition (%) = 100 - Cell viability (%).

#### Immunofluorescence identification of intracellular ZF9 protein

HEK-293T or Hela cells were seeded in 12-well plates at a density of 1×10^6^ cells per well for culturing overnight in 1000 μL of DMEM containing 10% FBS. After a 24 h incubation, the cells were detached and transferred into 1.5 mL Eppendorf tubes, then fixed with 4% paraformaldehyde and permeabilized with 0.3% Triton X-100. Alexa Flour 488 anti-His tag antibody was used at a dilution of 1:2000. The Alexa Flour 488 signal in the individual cells was analyzed by a BD FACSAria III flow cytometer. The cells displaying a normal morphology were gated first, and the green fluorescence was measured with a 488 nm laser. The percentage of cells exhibiting green fluorescence and the calculated mean fluorescent intensity (MFI) were recorded. In addition, Hela cells treated in the same way were microscopically imaged using the Opera Phenix (Perkin Elmer) to observe the nuclear distribution of the delivered proteins.

#### ZF-bound plasmid transfection *in vitro*

HEK-293T cells were seeded in 12-well plates at a density of 1×10^6^ cells per well for culturing overnight. Before the transfection experiment began, the medium was replaced with 900 μL of serum-free DMEM or DMEM supplemented with an indicated percent of FBS. For the binding of ZF protein and plasmids, 10 μg pTT5-tdTomato plasmids containing ZF-binding sites were added to 100 μL of 2 nmol eTAT-ZF recombinant proteins diluted in serum-free DMEM or DMEM supplemented with an indicated percent of FBS, and the mixture was incubated at room temperature for 15 min. Then the ZF9-containing mixture was added to the plated cells and incubated at 37°C for 3 h. Subsequently, the transfected cells were washed three times with serum-free DMEM containing 10 U/ml heparin and cultured in DMEM supplemented with 10% FBS.

For the control, a mixture of 1 μg pTT5-tdTomato plasmids and 3 μL X-tremeGENE was incubated at room temperature for 20 min and then added to plated cells cultured in 1 mL of freshly changed serum-free DMEM or DMEM supplemented with FBS at the indicated percentage. After incubation at 37°C for 8 h, the cell culture medium was changed with DMEM supplemented with 10% FBS.

Then, the cells transfected with ZF9-related proteins or X-tremeGENE were detached at the indicated time points post-transfection, and the percentages of red fluorescence-positive cells were recorded using a flow cytometer (LSRFortessa X-20 Cell Analyzer, BD). The gating strategy to sort tdTomato-positive cells in HEK-293T was presented in supplemental information (Figure S2). The data were collected by using BD FACSDiva software and analyzed by using FlowJo software.

#### ZF9-bound plasmid transfection *in vivo*

pTT5-luciferase plasmids (20 μg) containing ZF9-binding sites were added to 5 nmol ZF9-related recombinant proteins, and the mixture was incubated at room temperature for 15 min. The same volume (100 μL) of PBS served as the control. Then, 100 μL of each preincubated mixture or naked plasmid was injected intramuscularly into the musculus tibialis anterior of seven-week-old nude female BALB/c nude mice. After 48 h, D-luciferin (150 mg/kg) was administered intraperitoneally, and the luciferase expression was monitored by live-animal imaging (IVIS-Lumina II, PerkinElmer Z) 15-20 min post-luciferin injection.

For the SARS-CoV-2 nucleocapsid protein-encoding DNA immunizations, three groups of six female BALB/c nude mice were immunized by intramuscular injection delivery of the plasmid DNA encoding the SARS-CoV-2 nucleocapsid protein and 6×ZF9-binding sites (plasmid N). Group 1 received 4 injections with PBS, group 2 received 4 immunizations with 20 μg naked plasmid N, group 3 received 4 immunizations with the mixture of 20 μg plasmid N and 5 nmol TAT-ZF9-NLS recombinant protein, and group 4 received 4 immunizations with the mixture of 20 μg plasmid N and 5 nmol eTAT-ZF9-NLS recombinant protein. There was an interval of one week between each immunization. After the end of immunization, blood samples were collected from the orbital vein on days 0, 10, 20, and 30 after primary immunization, and serum antibody levels of nucleocapsid protein were detected by indirect ELISA after 1000 times dilution.

#### Biosafety analysis of eTAT-ZF9-NLS *in vivo* administration

The biosafety of BALB/c nude mice after administration of eTAT-ZF9-NLS was investigated. Mice were injected with the same dose of TAT-ZF9-NLS and plasmid mixture as *in vivo* transfection, and untreated mice and AAV-Luc administration groups were used as controls. Whole blood samples were collected from the orbital vein, and serum was isolated by centrifugation at 15000 g for 10 min. Then, the liver function was evaluated by determining the serum level of aminotransferase (ALT) and aminotransferase (AST) by using the ALT and AST assay kits (Jiancheng, Nanjing, China) (n=3), and the kidney function was evaluated by the level of kidney injury molecule-1 (KIM-1) by using the Mouse KIM-1 ELISA kit (Beyotime, Shanghai, China) (n=3). After euthanasia, the major organs (including heart, liver, spleen, lung, kidney) were processed for H&E staining according to standard H&E staining protocols for paraffin-embedded tissue sections with a thickness of 4 μm. Images were obtained at ×200 with a Zeiss Axio Scan and ZEN Imaging Software.

### Quantification and statistical analysis

GraphPad Prism software version 7.04 (GraphPad Software, Inc. San Diego, CA) was employed for the statistical analyses. The data are presented as the means ± s.e.m. Unpaired two-tailed Student’s t-tests were used to compare the differences between two groups. Differences in the compared groups were considered significantly different with P values less than 0.05. ∗, ∗∗, ∗∗∗ and ∗∗∗∗ indicate P values less than 0.05, 0.01, 0.001 and 0.0001, respectively.
