# Supplementary material for: A non-viral DNA delivery system consisting of multifunctional chimeric peptide fused with zinc-finger protein
Source: iScience. 2024 Mar 8;27(4):109464. doi: 10.1016/j.isci.2024.109464 (PMC10981093; doi:10.1016/j.isci.2024.109464)
Supplement: Document S1. Figures S1–S5 and Tables S1–S3 [file mmc1.pdf]

iScience, Volume *27*

Supplemental information

A non-viral DNA delivery system consisting of multifunctional chimeric peptide fused with zinc-finger protein

Siyuan Yu, Haifeng Pan, Han Yang, Haoyun Zhuang, Haihui Yang, Xuan Yu, Shiyin Zhang, Mujin Fang, Tingdong Li, Shengxiang Ge, and Ningshao Xia

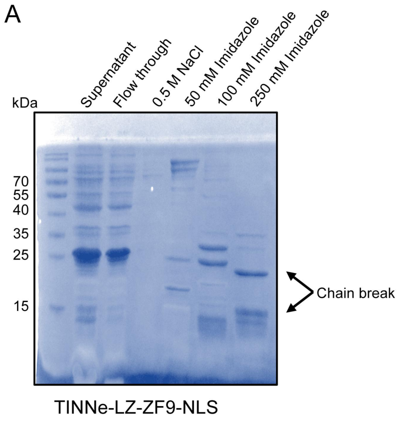

Fig. S1. The SDS-PAGE analysis of purified ZF9-related recombinant proteins, Related to Figure 1, Figure 2, and Figure 4. Molecular weight markers were loaded in the first or last line of both SDS-PAGEs. (A) SDS-PAGE analysis of the TINNe-LZ-ZF9-NLS protein fractions collected from the Ni-NTA affinity column. (B, C) SDS-PAGE analysis of ZF9-related recombinant proteins expressed in E.coli and purified on a Ni-NTA affinity column. Data shown in B and C are representative of two independent experiments, respectively.

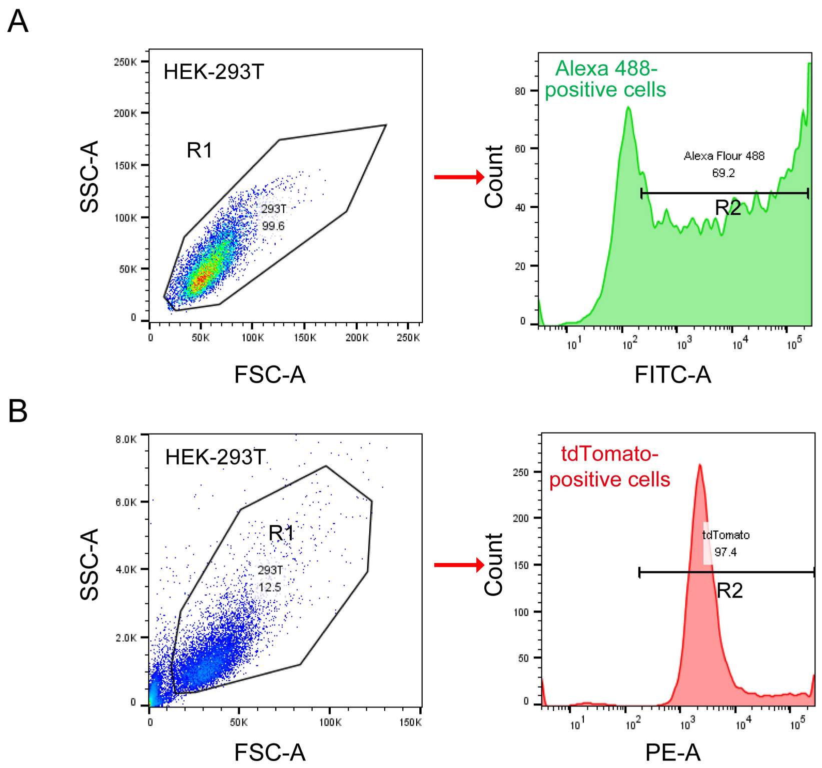

Fig. S2. Schematic of flow cytometry gating strategy, Related to Figure 1, Figure 2, Figure 3, and Figure 4. (A) Gating strategy to measure the mean fluorescence intensities (MFIs) of the HEK-292T cells treated with eTAT-ZF-NLS recombinant proteins and analyzed by flow cytometry using Alexa Flour 488 labeled anti-His tag antibody. The cells displaying a normal morphology were gated first (left panel, R1) and then the mean fluorescence green intensities within total HEK-293T cells were recorded. This strategy was used in the analysis of delivery efficiency presented in Figure 1d. (B) Gating strategy to sort tdTomato-positive cells in HEK-293T cells and other mammalian cells. Cells were first gated on morphology (left panel, R1), then the red fluorescent cells of R1 were analyzed and the percentage of tdTomato-positive cells was determined (right panel, R2). This strategy was used in the detection and quantification of tdTomato gene transfection efficiency presented in Figures 2B, 2D, 3B, 3D, 4A, and 4B.

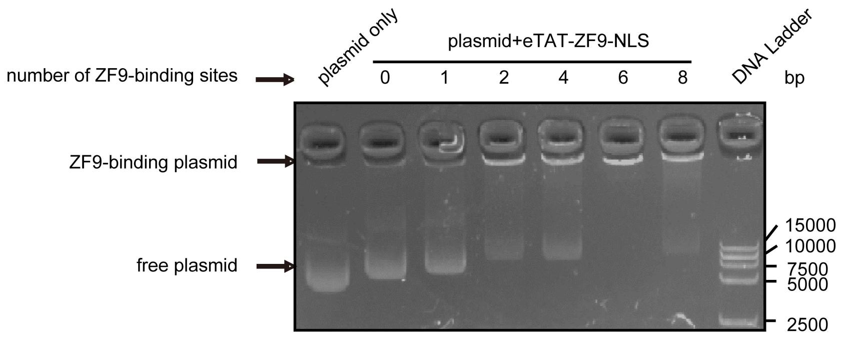

Fig. S3. Agarose gel electrophoresis of reporter plasmids mixed with eTAT-ZF9-NLS, Related to Figure 3. 2 μg of reporter plasmids containing different tandem repeats of ZF9-binding sites (0×, 1×, 2×, 4×, 6× or 8×) were mixed with 0.4 nmol eTAT-ZF9-NLS recombinant protein in a total volume of 30 μl, respectively. After 15 minutes of incubation, the mixture was subjected to a 1 % agarose gel electrophoresis analysis. The naked plasmid was loaded as a control.

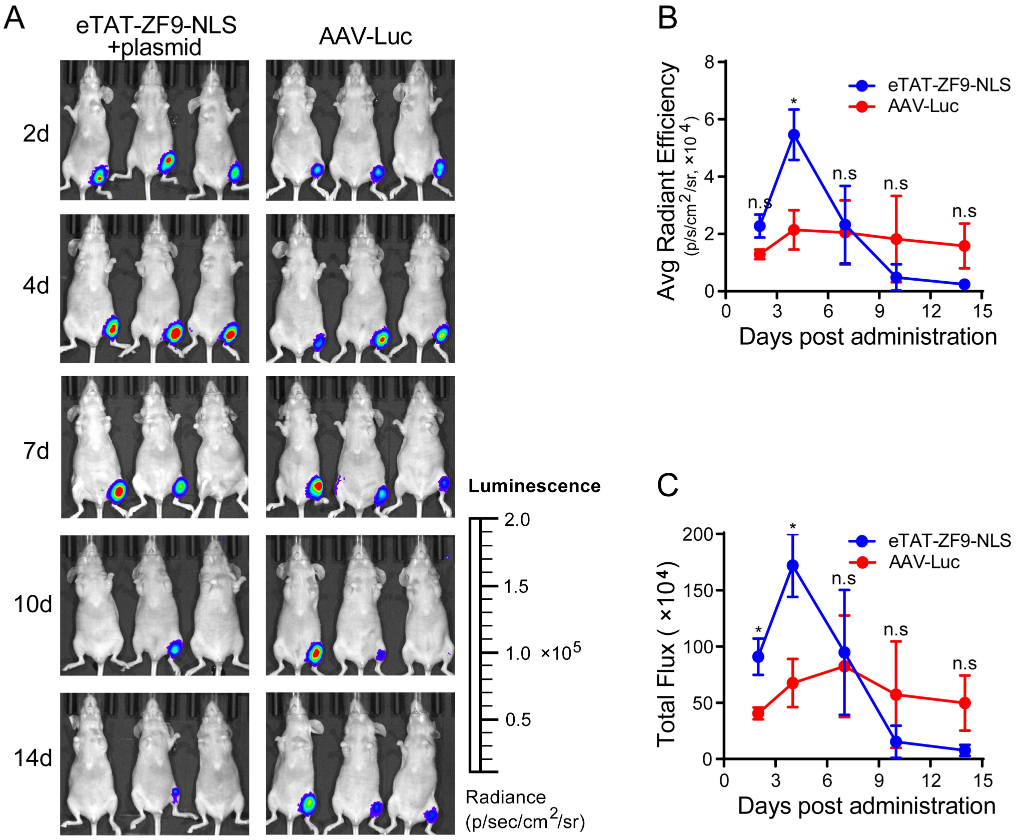

Fig. S4. The relative luciferase activities in the left hind leg of BALB/c nude mice, Related to Figure 5. The relative luciferase activities were monitored by (A) *in vivo* imaging and (B, C) ROI fluorescence intensity during 0 to 14 days after intramuscular injection of eTAT-ZF9-NLS mixed Luciferase reporter plasmid or AAV-Luc (n=3). The data are expressed as the means ± s.e.m., unpaired Student t-test, and P-value are shown, n.s: no significant difference.

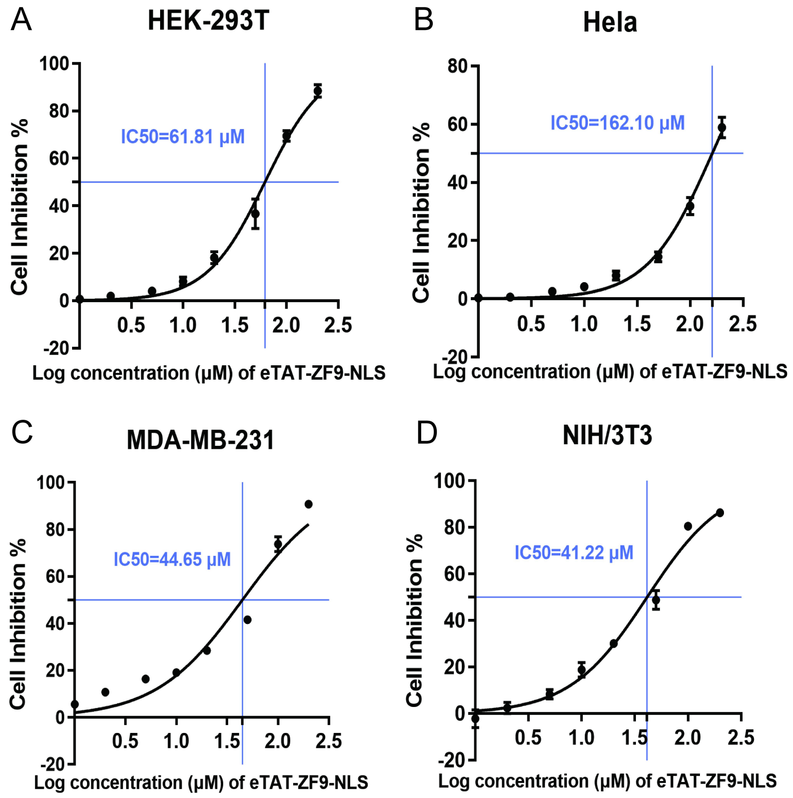

Fig. S5. The cytotoxicity curve and IC50 data of eTAT-ZF9-NLS for the representative cells, Related to Figure 3. (A) HEK-293T, (B) Hela, (C) MDA-MB-231, and (D) NIH/3T3 cells were treated with various concentrations of eTAT-ZF9-NLS protein for 24 hours. IC50 was calculated from the dose-response curve. Error bars represent SEMs from 3 independent measurements.

Tab. S1. The DNA sequence of The ZF proteins involved in the ZFs library and their specific binding sites, Related to Figure 1 and Figure 2.

Supplementary Table 1		
**ZF number**	**Bingding site 5'-3'**	**ZF sequence**
ZF1	aGGAGGGGCTc	GTCTCTAGACCCGGGGAGCGCCCCTTCCAGTGTCGCATTTGCATGCGGAACTTTTCGATGAAAAATACTTTGACTAGACATACCCGTACTCATACCGGTGAAAAACCGTTTCAGTGTCGGATCTGTATGCGAAATTTCTCCAGACAAGAACATTTGGTTAGACATCTACGTACGCACACCGGCGAGAAGCCATTCCAATGCCGAATATGCATGCGCAACTTCAGTCAAAAACCACATTTGTCAAGACACCTAAAAACCCACCTGAGAGGATCC
ZF2	gGCCGAAGATa	GTCTCTAGACCCGGGGAGCGCCCCTTCCAGTGTCGCATTTGCATGCGGAACTTTTCGACTGGTCAAAGATTGAGAATTCATACCCGTACTCATACCGGTGAAAAACCGTTTCAGTGTCGGATCTGTATGCGAAATTTCTCCCAAAATCAAAATTTGGCTAGACATCTACGTACGCACACCGGCGAGAAGCCATTCCAATGCCGAATATGCATGCGCAACTTCAGTGATAAATCTGTTTTGGCTAGACACCTAAAAACCCACCTGAGAGGATCC
ZF3	aGAGTGAGGAc	GTCTCTAGACCCGGGGAGCGCCCCTTCCAGTGTCGCATTTGCATGCGGAACTTTTCGCGCCAGGACAGGCTTGACAGGCATACCCGTACTCATACCGGTGAAAAACCGTTTCAGTGTCGGATCTGTATGCGAAATTTCTCCCAGAAGGAGCACTTGGCGGGGCATCTACGTACGCACACCGGCGAGAAGCCATTCCAATGCCGAATATGCATGCGCAACTTCAGTCGCCGCGACAACCTGAACCGGCACCTAAAAACCCACCTGAGAGGATCC
ZF4	cGGGGACGTCa	GTCTCTAGACCCGGGGAGCGCCCCTTCCAGTGTCGCATTTGCATGCGGAACTTTTCGACTGCTGCTGTTTTGACTAGACATACCCGTACTCATACCGGTGAAAAACCGTTTCAGTGTCGGATCTGTATGCGAAATTTCTCCGATAGAGCTAATTTGACTAGACATCTACGTACGCACACCGGCGAGAAGCCATTCCAATGCCGAATATGCATGCGCAACTTCAGTAGAATTGATAAATTGGGTGATCACCTAAAAACCCACCTGAGAGGATCC
ZF5	gGTGTAGGGGt	GTCTCTAGACCCGGGGAGCGCCCCTTCCAGTGTCGCATTTGCATGCGGAACTTTTCGAAAGGTGAAAGATTGGTTAGACATACCCGTACTCATACCGGTGAAAAACCGTTTCAGTGTCGGATCTGTATGCGAAATTTCTCCAGAATGGATAATTTGTCTACTCATCTACGTACGCACACCGGCGAGAAGCCATTCCAATGCCGAATATGCATGCGCAACTTCAGTAGAAAAGATGCTTTGAATAGACACCTAAAAACCCACCTGAGAGGATCC
ZF6	tGCAGGAGGTg	GTCTCTAGACCCGGGGAGCGCCCCTTCCAGTGTCGCATTTGCATGCGGAACTTTTCGATTCCAAATCATTTGGCTAGACATACCCGTACTCATACCGGTGAAAAACCGTTTCAGTGTCGGATCTGTATGCGAAATTTCTCCCAATCTGCTCATTTGAAAAGACATCTACGTACGCACACCGGCGAGAAGCCATTCCAATGCCGAATATGCATGCGCAACTTCAGTCAAGATGTTTCTTTGGTTAGACACCTAAAAACCCACCTGAGAGGATCC

ZF7	aGCTGGAGGGt	GTCTCTAGACCCGGGGAGCGCCCCTTCCAGTGTCGCATTTGCATGCGGAACT<br>TTTCGAAAAAAGATCATTTGCATAGACATACCCGTACTCATACCGGTGAAAA<br>ACCGTTTCAGTGTCGGATCTGTATGCGAAATTTCTCCCAAAGACCACATTTG<br>ACTAATCATCTACGTACGCACACCGGCGAGAAGCCATTCCAATGCCGAATAT<br>GCATGCGCAACTTCAGTGTTGGTGCTTCTTTGAAAAGACACCTAAAAACCC<br>ACCTGAGAGGATCC
ZF8	cGAAGTGGTCc	GTCTCTAGACCCGGGGAGCGCCCCTTCCAGTGTCGCATTTGCATGCGGAACT<br>TTTCGACTATGGCTGTTTTGAGAAGACATACCCGTACTCATACCGGTGAAAA<br>ACCGTTTCAGTGTCGGATCTGTATGCGAAATTTCTCCAGAAGAGAAGTTTTG<br>GAAAATCATCTACGTACGCACACCGGCGAGAAGCCATTCCAATGCCGAATAT<br>GCATGCGCAACTTCAGTCAAACTGTTAATTTGGATAGACACCTAAAAACCCA<br>CCTGAGAGGATCC
ZF9	tGTAGATGGAg	GTCTCTAGACCCGGGGAGCGCCCCTTCCAGTGTCGCATTTGCATGCGGAACT<br>TTTCGGATAAAACTAAATTGAGAGTTCATACCCGTACTCATACCGGTGAAAA<br>ACCGTTTCAGTGTCGGATCTGTATGCGAAATTTCTCCGTTAGACATAATTTGA<br>CTAGACATCTACGTACGCACACCGGCGAGAAGCCATTCCAATGCCGAATATG<br>CATGCGCAACTTCAGTCAATCTACTTCTTTGCAAAGACACCTAAAAACCCAC<br>CTGAGAGGATCC
ZF10	tGAAGAAGCTg	GTCTCTAGACCCGGGGAGCGCCCCTTCCAGTGTCGCATTTGCATGCGGAACT<br>TTTCGTCTGCTCAAGCTTTGGCTAGACATACCCGTACTCATACCGGTGAAAA<br>ACCGTTTCAGTGTCGGATCTGTATGCGAAATTTCTCCCAAGGTGGTAATTTG<br>ACTAGACATCTACGTACGCACACCGGCGAGAAGCCATTCCAATGCCGAATAT<br>GCATGCGCAACTTCAGTCAACATCCAAATTTGACTAGACACCTAAAAACCC<br>ACCTGAGAGGATCC

Tab. S2. The DNA sequence of the main components, Related to Figure 1. The DNA sequence of the main components of the eTAT-ZF9-NLS transfection system and the reporter gene used to evaluate the transfection efficiency *in vitro* and *in vivo*. TAT, INF7, N/Ne and CC-Tri3 were the components of eTAT chimaeric peptide. Each DNA sequence is briefly described.

Supplementary Table 2		
**Name of sequence**	**Sequence 5'-3'**	**Comment**
TAT	GGCAGGAAGAAGCGGAGACAGCGACGAAGACCGCCGCAG	Cell-penetrating peptide TAT, derived from the HIV Tat protein (amino acids 48-60), has the ability to mediate biological macromolecules across the cell membrane to achieve intracellular delivery.
INF7	GGCCTGTTCGAAGCAATAGAAGGTTTCATAGAAAATGGTTGG GAGGGAATGATAGACGGTTGGTACGGT	pH-Dependent Membrane Active Peptide INF7, a synthetic derivative of the amino terminal fusion peptide of influenza hemagglutinin HA-2 subunit. Because it can only have liposome membrane leakage activity at lower pH, it is often used in endosome (pH 5.5~6.5) disruption to promote cargo escape.
N	AACAACACTCATGACCTTGTCGGTGATGTGAGATTAGCCGGA GTT	A protease cleavage site sequence at the N-terminus of the F protein of Nipah virus (amino acids 99-113), has been verified to be efficiently cleaved by cathepsin L (CTSL). In this study, it was used to release the protein cargo from the endosome into the cytosol.
Ne	CAGAGCGTTGCAAGCAGCCGTCGTCATAAACGTTTTGCAGGT GTT	A protease cleavage site sequence at the N-terminus of the F protein of the measles virus (amino acids 102-116), has been verified to be efficiently cleaved by Furin. In this study, it was used to release the protein cargo from the endosome into the cytosol.
CC-Tri3	GAAATTGCAGCAATTAAAAAAGAAATTGCAGCAATTAAACAG GAAATTGCAGCAATTAAACAGGGTTATGGT	An artificially designed coiled-coil motif with a length of about 20 amino acid residues that can spontaneously form a homotrimer. In this study, it was verified that it can be fused with other proteins at the amino-terminal to make the recombinant protein form a stable homodimer.
NLS	CCCAAGAAGAAGCGTAAGGTG	A single nuclear localization signal (NLS) is described as a cluster composed of 3-5 basic residues, of which the SV40 large T antigen NLS is recognized as the prototype sequence, PKKKRKV. Additional SV40 NLS fusion has been found to increase the ability of proteins to enter the nucleus. In this study, NLS was fused to the carboxyl

		terminus of ZF9 to promote plasmid entry into the nucleus.
6×His Tag	CACCACCACCACCACCAC	6×His tag was added to the C-terminus of the recombinant protein for affinity purification
tdTomato	ATGGTGAGCAAGGGCGAGGAGGTCATCAAAGAGTTCATGCGCTTCAAGGTGCGCATGGAGGGCTCCATGAACGGCCACGAGTTCGAGATCGAGGGCGAGGGCGAGGGCCGCCCCTACGAGGGCACCCAGACCGCCAAGCTGAAGGTGACCAAGGGCGGCCCCCTGCCCTTCGCCTGGGACATCCTGTCCCCCCAGTTCATGTACGGCTCCAAGGCGTACGTGAAGCACCCCGCCGACATCCCCGATTACAAGAAGCTGTCCTTCCCCGAGGGCTTCAAGTGGGAGCGCGTGATGAACTTCGAGGACGGCGGTCTGGTGACCGTGACCCAGGACTCCTCCCTGCAGGACGGCACGCTGATCTACAAGGTGAAGATGCGCGGCACCAACTTCCCCCCCGACGGCCCCGTAATGCAGAAGAAGACCATGGGCTGGGAGGCCTCCACCGAGCGCCTGTACCCCCGCGACGGCGTGCTGAAGGGCGAGATCCACCAGGCCCTGAAGCTGAAGGACGGCGGCCACTACCTGGTGGAGTTCAAGACCATCTACATGGCCAAGAAGCCCGTGCAACTGCCCGGCTACTACTACGTGGACACCAAGCTGGACATCACCTCCCACAACGAGGACTACACCATCGTGGAACAGTACGAGCGCTCCGAGGGCCGCCACCACCTGTTCCTGGGGCATGGCACCGGCAGCACCGGCAGCGGCAGCTCCGGCACCGCCTCCTCCGAGGACAACAACATGGCCGTCATCAAAGAGTTCATGCGCTTCAAGGTGCGCATGGAGGGCTCCATGAACGGCCACGAGTTCGAGATCGAGGGCGAGGGCGAGGGCCGCCCCTACGAGGGCACCCAGACCGCCAAGCTGAAGGTGACCAAGGGCGGCCCCCTGCCCTTCGCCTGGGACATCCTGTCCCCCCAGTTCATGTACGGCTCCAAGGCGTACGTGAAGCACCCCGCCGACATCCCCGATTACAAGAAGCTGTCCTTCCCCGAGGGCTTCAAGTGGGAGCGCGTGATGAACTTCGAGGACGGCGGTCTGGTGACCGTGACCCAGGACTCCTCCCTGCAGGACGGCACGCTGATCTACAAGGTGAAGATGCGCGGCACCAACTTCCCCCCCGACGGCCCCGTAATGCAGAAGAAGACCATGGGCTGGGAGGCCTCCACCGAGCGCCTGTACCCCCGCGACGGCGTGCTGAAGGGCGAGATCCACCAGGCCCTGAAGCTGAAGGACGGCGGCCACTACCTGGTGGAGTTCAAGACCATCTACATGGCCAAGAAGCCCGTGCAACTGCCCGGCTACTACTACGTGGACACCAAGCTGGACATCACCTCCCACAACGAGGACTACACCATCGTGGAACAGTACGAGCGCTCCGAGGGCCGCCACCACCTGTTCCTGTACGGCATGGACGAGCTGTACAAG	Used as a fluorescent reporter to monitor transfection efficiency *in vitro*
Luciferase	ATGGAAGACGCCAAAAACATAAAGAAAGGCCCGGCGCCATTCTATCCTCTTGAGGATGGAACCGCTGGAGAGCAACTGCATAAGGCTATGAAGAGATACGCCCTGGTTCCTGGAACAATTGCTTTTACAGATGCACATATCGAGGTGAACATCACGTACGCGGAATACTT	Used to determine the transfection efficiency *in vivo*

	CGAAATGTCCGTTCGGTTGGCAGAAGCTATGAAACGATATGGG<br>CTGAATACAAATCACAGAATCGTCGTATGCAGTGAAAACTCTC<br>TTCAATTCTTTATGCCGGTGTTGGGCGCGTTATTTATCGGAGTT<br>GCAGTTGCGCCCGCGAACGACATTTATAATGAACGTGAATTGC<br>TCAACAGTATGAACATTTCGCAGCCTACCGTAGTGTTTGTTTC<br>CAAAAAGGGGTTGCAAAAAATTTTGAACGTGCAAAAAAAATT<br>ACCAATAATCCAGAAAATTATTATCATGGATTCTAAAACGGATT<br>ACCAGGGATTTCAGTCGATGTACACGTTCGTCACATCTCATCT<br>ACCTCCCGGTTTTAATGAATACGATTTTGTACCAGAGTCCTTTG<br>ATCGTGACAAAACAATTGCACTGATAATGAACTCCTCTGGATC<br>TACTGGGTTACCTAAGGGTGTGGCCCTTCCGCATAGAACTGCC<br>TGCGTCAGATTCTCGCATGCCAGAGATCCTATTTTTGGCAATCA<br>AATCATTCCGGATACTGCGATTTTAAGTGTTGTTCCATTCCATC<br>ACGGTTTTGGAATGTTTACTACACTCGGATATTTGATATGTGGA<br>TTTCGAGTCGTCTTAATGTATAGATTTGAAGAAGAGCTGTTTTT<br>ACGATCCCTTCAGGATTACAAAATTCAAAGTGCGTTGCTAGTA<br>CCAACCCTATTTTCATTCTTCGCCAAAAGCACTCTGATTGACA<br>AATACGATTTATCTAATTTACACGAAATTGCTTCTGGGGGCGCA<br>CCTCTTTCGAAAGAAGTCGGGGAAGCGGTTGCAAAACGCTTC<br>CATCTTCCAGGGATACGACAAGGATATGGGCTCACTGAGACTA<br>CATCAGCTATTCTGATTACACCCGAGGGGGATGATAAACCGGG<br>CGCGGTCGGTAAAGTTGTTCCATTTTTTGAAGCGAAGGTTGTG<br>GATCTGGATACCGGGAAAACGCTGGGCGTTAATCAGAGAGGC<br>GAATTATGTGTCAGAGGACCTATGATTATGTCCGGTTATGTAAA<br>CAATCCGGAAGCGACCAACGCCTTGATTGACAAGGATGGATG<br>GCTACATTCTGGAGACATAGCTTACTGGGACGAAGACGAACA<br>CTTCTTCATAGTTGACCGCTTGAAGTCTTTAATTAAATACAAAG<br>GATACCAGGTGGCCCCCGCTGAATTGGAGTCGATATTGTTACA<br>ACACCCCAACATCTTCGACGCGGGCGTGGCAGGTCTTCCCGA<br>CGATGACGCCGGTGAACTTCCCGCCGCCGTTGTTGTTTTGGAG<br>CACGGAAAGACGATGACGGAAAAAGAGATCGTGGATTACGTC<br>GCCAGTCAAGTAACAACCGCGAAAAAGTTGCGCGGAGGAGT<br>TGTGTTTGTGGACGAAGTACCGAAAGGTCTTACCGGAAAACT<br>CGACGCAAGAAAAATCAGAGAGATCCTCATAAAGGCCAAGAA<br>GGGCGGAAAGTCCAAATTG	

Tab. S3. Different ZF9 fusion proteins were used to evaluate the transfection efficiency of the reporter plasmid, Related to Figure 4.

Supplementary Table 3			
**Short name**	**Full name**	**Composition**	**Amino acid sequence**
T-ZF9-NLS	TAT-ZF9-NLS	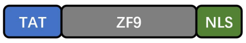	MGRKKRRQRRRPPQVSRPGERPFQCRICMRNFS<br>DKTKLRVHTRTHTGEKPFQCRICMRNFSVRHNLT<br>RHLRTHTGEKPFQCRICMRNFSQSTSLQRHLKTH<br>LRGSPKKKRKVHHHHHH
TI-ZF9-NLS	TAT-INF7-ZF9-N<br>LS	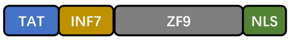	MGRKKRRQRRRPPQGLFEAIEGFIENGWEGMID<br>GWYGVSRPGERPFQCRICMRNFSDKTKLRVHTR<br>THTGEKPFQCRICMRNFSVRHNLTRHLRTHTGEK<br>PFQCRICMRNFSQSTSLQRHLKTHLRGSPKKKRK<br>VHHHHHH
TINNe-ZF9-NLS	TAT-INF7-NNe-Z<br>F9-NLS	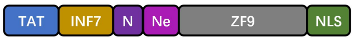	MGRKKRRQRRRPPQGLFEAIEGFIENGWEGMID<br>GWYGGGGGSNNTHDLVGDVRLAGVGGGGSQSV<br>ASSRRHKRFAGVGGGGSVSRPGERPFQCRICMR<br>NFSDKTKLRVHTRTHTGEKPFQCRICMRNFSVRH<br>NLTRHLRTHTGEKPFQCRICMRNFSQSTSLQRHL<br>KTHLRGSPKKKRKVHHHHHH
eTAT-ZF9-NLS	eTAT-ZF9-NLS	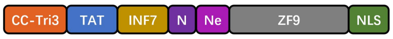	MEIAAIKKEIAAIKQEIAAIKQGYGGGGGSGRKK<br>RRQRRRPPQGLFEAIEGFIENGWEGMIDGWYGG<br>GGGSNNTHDLVGDVRLAGVGGGGSQSVASSRR<br>HKRFAGVGGGGSVSRPGERPFQCRICMRNFSDK<br>TKLRVHTRTHTGEKPFQCRICMRNFSVRHNLTRH<br>LRTHTGEKPFQCRICMRNFSQSTSLQRHLKTHLR<br>GSPKKKRKVHHHHHH
